# A Unifying Theory of Biological Function

**DOI:** 10.1007/s13752-017-0261-y

**Published:** 2017-03-08

**Authors:** J. H. van Hateren

**Affiliations:** Johann Bernoulli Institute for Mathematics and Computer Science, University of Groningen, Groningen, The Netherlands

**Keywords:** Agency, Evolution, Function, Goal-directedness, Normativity

## Abstract

A new theory that naturalizes biological function is explained and compared with earlier etiological and causal role theories. Etiological (or selected effects) theories explain functions from how they are caused over their evolutionary history. Causal role theories analyze how functional mechanisms serve the current capacities of their containing system. The new proposal unifies the key notions of both kinds of theories, but goes beyond them by explaining how functions in an organism can exist as factors with autonomous causal efficacy. The goal-directedness and normativity of functions exist in this strict sense as well. The theory depends on an internal physiological or neural process that mimics an organism’s fitness, and modulates the organism’s variability accordingly. The structure of the internal process can be subdivided into subprocesses that monitor specific functions in an organism. The theory matches well with each intuition on a previously published list of intuited ideas about biological functions, including intuitions that have posed difficulties for other theories.

## Introduction

Many of the parts and processes of biological organisms appear to have functions. For example, pumping blood appears to be the primary function of the heart, and enabling vision appears to be the primary function of the eye. The concept of function has several interpretations (Wright 1973), but at least some of these seem to imply an implicit goal-directedness. The heart is expected to pump blood, and it has properties that are well suited to that end. There is often also a valuative, normative aspect to functions, because a properly functioning heart seems good for an organism and a malfunctioning one seems bad. Both goal-directedness and normativity are puzzling, because they do not occur in the non-living parts of nature. One may therefore wonder if and how they can arise in living organisms.

In this article, I will analyze biological functions from a naturalistic perspective. Thus, I assume that they can be understood as being produced by basic, physicochemical processes. I will show that functions can be autonomous causal factors, not depending on human understanding. This also applies to their goal-directedness and normativity. I will not perform a detailed conceptual analysis of the term “function”—neither an analysis of how it is typically used in natural languages, nor of how it is typically used by biologists studying functions. Approximate agreement between the concept of function developed here and typical usage is expected, but it is not a specific requirement or goal. The goal is to explain the ontology of functions, including their goal-directedness and normativity.

This article focuses on biological functions in non-human species. The reason for this restriction is that the analysis of biological functions in humans is complicated by the dual role humans have. They are biological organisms with functions of their own, but they are also the ones doing the interpretation of functions. Human sociality further complicates matters, because goals may become widely shared with others, which diffuses the benefits of a particular function. Although it is possible to extend the present approach to human functions, this is left to a future study. The same applies for an extension to the function of artifacts.

### Are Functions Epistemological Constructs or Ontological Causal Factors?

It is clear that the material structures that perform a function, for example the heart and its muscles and valves, are ontological causal factors, or at least are fully composed of such factors. These material structures produce their effects in the standard way of any physicochemical process. However, it is less clear what causal status one should assign to the function as such, for example, the function of pumping blood. If the function as such has no causal efficacy beyond that of its material realization, then it should be regarded as an epistemological construct. It may be real (pumping blood is real), but the function ascription would not need to be included in a complete and sufficient causal inventory of the world. Including the material realization of the function would suffice for that. On the other hand, if a function as such has causal efficacy that goes beyond that of its material realization, then it should be regarded as an ontological causal factor. A causal inventory of the world would not be complete without it.

This distinction between the ontology and epistemology of functions is used extensively below. Functions that possess autonomous causal efficacy are denoted by the term *ontic-causal*. “Ontic” is meant here to denote that such functions exist independently of whether human intellect (or equivalent) exists. “Causal” denotes that they are embedded in the causal dynamics of the world and that they form an autonomous and indispensable part of that dynamics. Functions that lack autonomous causal efficacy are denoted by the term *epistemic-real*. “Epistemic” means here that the perception of humans (or other life forms) is required for noting the material structure associated with such functions. “Real” denotes that this structure is still objective. It is neither subjective, nor disputable, nor dependent on the attitude of observers.

A standard physicalist view assumes that all material processes are completely defined by the underlying, fundamental physical processes. In that view, biological functions would be epistemic-real only, by definition. Moreover, their apparent goal-directedness and normativity would be epistemic-real as well. However, recent theoretical and computational work (van Hateren 2015a) has shown that goal-directedness is not necessarily epistemic-real. It can become ontic-causal through a subtle combination of deterministic and random processes, if this combination is subject to sustained evolution by natural selection. The structure of this theory is such that it can explain how ontic-causal functions can arise. In the next section, the theory is explained and applied to biological functions. Subsequently, other theories of biological function are discussed with respect to the question whether they produce epistemic-real or ontic-causal functions. It is argued that these theories produce epistemic-real functions only. Nevertheless, many of the key properties of these theories transfer to the new theory, which can thus be seen as a unifying one. Finally, it is shown that the theory is consistent with an existing list of intuitions about functions (Wouters 2005).

## Explanation of the New Theory of Functions

The new theory of biological function is based on recent computational work (van Hateren 2015a). It conjectures that all living organisms contain an internal process X that approximates (i.e., estimates) the evolutionary fitness of the organism itself. This process subsequently modulates the variability of the organism in such a way that the actual fitness is likely to increase, on average. Below, I explain the theory qualitatively. First, I explain how fitness is defined here; second, how X can be understood; third, how X is thought to affect the organism; and, finally, why X thus acts as an emergent ontic-causal factor that can produce the ontic-causal status of biological functions.

The concept of fitness that is used here is a form of individual fitness (i.e., the fitness of an individual organism), not trait fitness (for a discussion of the difference see, e.g., Walsh 2015, Chap. 2). Moreover, fitness as used here is not the actual reproductive output of an organism. Instead, it is a propensity, that is, the organism’s capacity and tendency to survive and reproduce. Thus, it is not a post hoc measure of an organism’s actually realized success, but a concurrent measure of an organism’s likely success. The tendency to survive includes not only resilience against external threats, such as competitors and predators, but also the capacity for self-maintenance, such as through a well-functioning metabolism. The actually realized survival and reproduction relate to fitness only in a statistical sense, because they depend partly on random factors.

Fitness, as the capacity and tendency to survive and reproduce, is produced by a large range of factors that originate from the environment and from within the organism. For example, fitness is lower at times when food is scarce, because such scarcity decreases the organism’s chances of surviving and reproducing. Internal factors, such as malfunctioning internal organs, have similar effects. But fitness can recover when conditions improve. It becomes zero when the organism dies. In other words, fitness is a variable that changes continuously over time.

All factors that affect fitness can be conceived of as forming a highly complex fitness process, F. F is the totality of influences and processes that actually produce fitness (which is denoted by f, the organism’s tendency to survive and reproduce). It is important to understand that both F and f are epistemic-real constructs. The process F is just a standard physicochemical process, and thus is causally effective only through the microscopic factors of which it is composed. Neither F nor f have autonomous causal efficacy, that is, causal efficacy that goes beyond that of their composing factors (including how they interact). Another point that should be noted is that fitness as used here focuses on the organism, as the natural reproductive unit. However, the approach is not committed to a particular level of selection. Fitness depends on the entire process F producing the organism’s tendency to survive and reproduce. F includes organismal factors and factors arising from the physical environment. But it also includes population-level feedbacks, such as the Malthusian factor. This factor reduces the fitness of all organisms in a population when the population size approaches the environmental carrying capacity (e.g., when food or space becomes scarce). Frequency-dependent effects, such as those occurring in mimicry, are automatically included in F as well. Factors at a level below that of the organism, such as developmental and genetic ones, are also included. The approach is therefore, intrinsically, a multilevel one with respect to natural selection (i.e., differential reproduction). It does not assume, a priori, that any level of selection is more important than another one. This also applies to the mechanisms that can sustain traits across evolutionary time. Evolution by natural selection depends on the existence of such mechanisms. Although the most obvious mechanism is genetic, there are significant additional ones (e.g., epigenetics, the retention of cellular structures, niche construction, and social transmission).

The theory to be explained below conjectures that all living organisms contain an internal process X, with an output value x that approximates (i.e., estimates) the evolutionary fitness f of the organism itself. It is important to understand that x is not a kind of fitness, but a fitness approximation. The term “approximation” is used here and below as similar to estimate, estimation, simulated result, and proxy. It implies that the value of x should at least roughly reflect the value of f, similarly to how the reading of a thermometer should roughly reflect the actual temperature of the medium measured. But the quality of the approximation (estimation) could vary from poor to excellent. The processes X and F are very different entities, in the same sense that a weather simulation (made through observation and computation) is qualitatively (i.e., categorically) different from the weather itself. Both X and x are taken to be distributed throughout the organism, similarly to how that happens in a neural network. The physiological realization of X depends on the species. In unicellular organisms, it is fully realized by intracellular processes, such as those involved in sensing, computing, and acting. In multicellular organisms without extended nervous systems (e.g., plants), the process also involves physiological mechanisms for intercellular communication and regulation. In organisms with brains, much of X is thought to be realized by sensory and neural processing.

The existence of X is a theoretical conjecture, not a conjecture based on direct empirical indications of its existence. It is a distributed process that produces its effects through modulation of randomness (as explained below). Therefore, to observe or infer it by chance or serendipity would be extraordinarily difficult. Causally effective random variability is very hard to observe when it is embedded in deterministic causal processes, which are either dominant or assumed to be dominant. The existence of X can, then, only be established by well-designed, targeted experiments. But in order to do such experiments one already has to know what to look for. In other words, theory has to lead observation in this particular case.

Nevertheless, it is plausible that an X process can be present, given current knowledge of (neuro)physiology. Organisms routinely monitor many internal and external variables that affect their fitness. For example, a unicellular organism monitors the presence of nutrients surrounding it. Organisms contain physiological or neural circuits that can respond to adverse or beneficial conditions if these are indicated by such monitoring. For example, an organism may respond by moving to a different place or by switching to a different kind of nutrient. Such responses are typically made in primarily deterministic ways, as part of conventional cybernetic control circuits (not unlike the ones used in systems engineering and robotics). However, the circuits that detect adverse or beneficial conditions can play a dual role by also participating in the X process. The response produced by this process is not deterministic at all, but purely in the form of modulating random variability. Nevertheless, X does not need much additional circuitry for being present, because it can piggyback on existing molecular, cellular, and neural circuitry. Metaphorically speaking, it would be a fuzzy, stochastic process that is interwoven with the more easily observed deterministic processes.

The main effect of X, modulation of randomness, is a plausible mechanism as well. Physiology and neurophysiology are based on molecular processes, which are intrinsically highly variable (mainly because of the thermal variability that is inevitable when the number of molecules is small). Such variability is detrimental for the working of many biological subsystems. Thus, a large range of mechanisms exist that specifically reduce variability (e.g., DNA proofreading and repair, intracellular molecular amplification, and averaging over time and space by sensory and neural processes; see, e.g., Faisal et al. 2008). Varying the engagement of such variation-reducing mechanisms readily produces the type of modulation of variability required by the theory explained below. In other words, variability is typically controlled already, and modulating variability just requires controlling the control.

**Fig. 1:**
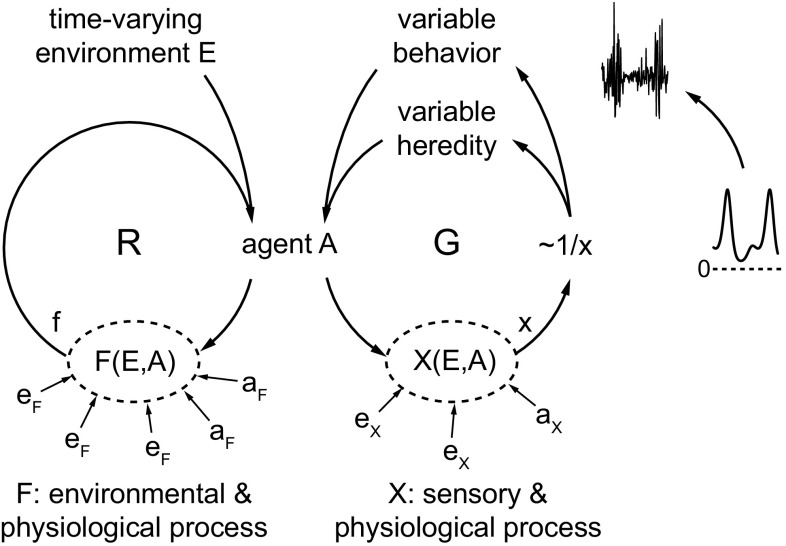
The new theory assumes an internalized approximation of fitness, x (produced by a process X), of the actual fitness f (produced by a process F). See the main text for further explanation

How X can affect an organism in illustrated in Fig. 1. The leftmost loop, R, symbolizes the basic process of evolution by natural selection. Organisms (abbreviated to “agents” here and below) reproduce, on average, in proportion to their fitness, f. The basic R loop thus produces evolution by differential reproduction. Agents with higher f than others reproduce more, on average. Thereby, they tend to increase the contribution of their hereditary traits to future populations. This gradually changes the likelihood that specific traits occur in agents, or, equivalently, gradually changes the distribution of traits over a population of agents. As explained above, f is the result of a fitness process F, which depends on factors a_F_ coming from within an agent A (e.g., related to its actual physiological state) and on factors e_F_ coming from outside an agent (summarized as A’s environment E). Factors e_F_ are related to actual physical and biological circumstances, such as the presence of food and mates.

In addition to this basic effect of fitness, fitness is conjectured to influence evolution through a secondary path (van Hateren 2015a). This is symbolized by the G loop in Fig. 1. As explained above, each agent is assumed to incorporate a process X of which the output x approximates f. X is also produced by many composing factors—a_X_ coming from within the agent and e_X_ coming from the environment. However, the factors a_X_ and e_X_ are observed and processed by the agent, using sensors and implicit physiological and neural computations. Thus, they are not identical to the actual corresponding factors a_F_ and e_F_, but, at best, approximations of those factors. Again, a_X_ and e_X_ are as different from a_F_ and e_F_ as an observed temperature used in a weather simulation is—categorically—different from the actual physical temperature that participates in producing the weather. The factors a_X_ and e_X_ are included in X typically because previous selection has established them as probably being important for approximating f. For example, a bacterium may sense the presence of glucose in its surroundings and may monitor its internal nutritional state. These factors are processed by X together with a large range of other factors, which results in an x that approximates f.

The resulting x is assumed to drive random variability in the structure of the agent, producing change at two different timescales. First, variability of heredity (through recombination, mutation, and epigenetics) affects consecutive agents in a lineage. This produces change at the timescale of evolution (van Hateren 2015b). Second, variability of behavior and behavioral dispositions affects the agent, but usually not its lineage. This produces change at the timescale of an agent’s lifetime. The term “behavior” should be interpreted very broadly here. It includes development, learning, and phenotypic plasticity in its widest sense. It also includes internal physiological changes within unicellular organisms and plants. Computational and mathematical analysis shows that the conjectured mechanism is evolvable if the variability is related to x in an inverse way (van Hateren 2015a, c). This is symbolized by the ~1/x in Fig. 1. The insets at the far right illustrate this point. When x is small (indicating a small fitness f), then 1/x is large (corresponding to the peaks in the lower diagram) and the resulting variability is large as well (corresponding to a high noise level in the upper diagram).

Intuitively, the working of the mechanism can be understood as follows. The explanation given below focuses on the timescale of an individual agent. Where appropriate, the case of an evolutionary timescale is added between parentheses, following “or,”. When x is large, the agent (or, its lineage) is probably doing well, and little variation is needed. But when x is small, the agent (or, its lineage) is probably not doing well, because low x indicates low fitness f. If nothing is changed, the agent may die (or, its lineage may become extinct). Part of the required change may be “known” to the agent if it is stored in physiological and neural circuits that were modified by previous learning. It may also be known to the agent (or, to the agents in a lineage) if it is stored in DNA that was modified by previous natural selection. Such known change can be executed automatically, as standard, mostly deterministic cybernetic control. It is not directly involved in the mechanism considered here.

However, in general, there is also unforeseeable change. Then neither the form of the change itself, nor the consequences it might have for fitness, is known. Such unknown change should be large when x is small (which indicates low fitness), because that increases the probability that behavior (or, heredity) with better x can be found. This follows from the fact that the G loop is continually active within an agent (or, that a sequence of G loops is continually active in a lineage). Continual cycling through the G loop (or, the sequence of G loops) will sooner or later happen to produce a large x, unless the agent perishes before that happens. When a large x is encountered, subsequent variability is reduced, because of ~1/x. Then the behavior of the agent (or, the heredity of the agent at that specific point in the lineage) will remain close to forms producing this large x. After some time (or, across generations), environmental change is likely to lower x. Then variability rises because ~1/x gets larger, and the search for high x starts all over again. Computations show that this mechanism is evolvable, at the behavioral timescale as well as at the evolutionary one (van Hateren 2015a). Populations of agents containing the mechanism outcompete populations lacking it (i.e., populations that utilize a fixed, but optimized, variability that is not modulated by x). The mechanism works because the low probability of successful change is compensated, on average, by the (probabilistic) prospect of fast, exponential growth in numbers. This growth follows when x (and thus f) is high enough. Even if many organisms will perish, the remaining ones can thrive and multiply.

The mechanism of the G loop is remarkable, because it utilizes randomness in a way that thoroughly mixes random and deterministic factors. In effect, it transfers determinacy by varying randomness. The outcome of a hereditary or behavioral trajectory that results from many G loop cycles is, therefore, unpredictable in detail. Yet, the long-term course of a trajectory is not completely random, because it is driven by x. At the behavioral timescale, such a combination of indeterminate and determinate factors is the signature of agency (van Hateren 2015a; agency is taken here as the ability to initiate behavior that is significant to the agent itself). In effect, the mechanism provides the agent with some behavioral freedom.

The agent (or, its lineage) appears to be driven into the direction of high x and thus, quite probably, high f as well. But this happens without explicit, foreseen directionality. The directionality is purely the statistical consequence of a random, probabilistic process. The G loop lets the behavior of the agent (or, the heredity of the agents in a lineage) drift away quickly from structures with low x, because low x produces large behavioral (or, hereditary) variability. In effect, the agent’s behavior (or, its heredity) accumulates at structures with high x, where variability, and thus drift, is low.

X is part of the agent and it originates within the agent through random modifications. Modifications of X affect fitness, through which they are, implicitly, evaluated. Importantly, the ultimate causal efficacy of X depends on the condition that x approximates fitness. This approximating relationship between x and f (i.e., the fact that x is an estimate of f) is in fact an emergent factor with autonomous causal efficacy. It has causal efficacy in addition to the direct (proximate) causal efficacy of the material parts of X. In particular, the model implies that the material parts of X can only affect fitness if the non-material relation between x and f is present as well. The latter is partly independent of X, because the relation not only depends on x, but also on F and f (which can vary autonomously and, to some extent, randomly). Therefore, both causal aspects of X are needed in conjunction, and they can be regarded as complementary. They produce neither epiphenomenalism, nor causal overdetermination.

The autonomous causal efficacy of the relation between x and f gives an ontic-causal status to x. Its relation with f needs to be included in a complete and minimal causal inventory of the world. As stated above, f itself is an epistemic-real construct that is fully defined by its microscopic constituents and their interactions. Readers may be puzzled by the fact that x obtains ontic-causal status by being related to an epistemic-real f. However, one should realize that the relation between x and f is not based on a regular physicochemical connection. Rather, it is an approximating relationship that cannot be defined in terms of physicochemical constituents. Properties of f do not transfer to x, just like the properties of the weather (e.g., that it is wet, hot, cold, or windy) do not physically transfer to a weather simulation. The weather and its simulation belong to different categories.

The approximating relationship between x and f is an emergent, nonmaterial factor with causal efficacy. The drive towards high x must be regarded, then, as the implicit goal of the agent (van Hateren 2015a). The agent combines this goal-directedness with the behavioral freedom provided by agency. Agency makes it possible that the agent changes its behavior in a direction away from the goal (i.e., towards lower x), even though changing in a direction towards the goal remains more likely. The strength of attraction towards the goal must be equated, then, to the value that the agent implicitly attaches to the goal. The goal of high x is implicitly normative, for the agent itself (van Hateren 2015d). The agent is expected to strive for high x, intrinsically. It is supposed to strive for high x not from the point of view of any external agent, but from the point of view of the agent itself. Thus, the G loop produces primordial forms of agency, goal-directedness, and normativity, as emergent factors. Moreover, it also produces a primordial form of causally effective reference, because X is causally effective only because x implicitly refers to f (in the form of an approximating relationship). Whereas reference plays no causal role in abiotic nature, it is present in systems if (and probably only if) these contain an X process. Because X presupposes evolution, such systems must be living organisms.

As argued above, high x must be regarded as the overall goal of an agent. But in practice, the process X is decomposed into subprocesses that serve specific subgoals, such as having a well-functioning heart, finding food, and finding mates. Together, these subprocesses and subgoals contribute to X and x. The intrinsic goals of the agent are completely defined by X. New goals are, by definition, incorporated into an accordingly changed X. Because X has a nonmaterial causal aspect (in the form of the relation between x and f), its subprocesses also have a nonmaterial causal aspect (in the form of the relation between their subgoals and the corresponding parts of F). Subprocesses that monitor specific functions then produce a causal efficacy that goes beyond that of the material realization of the functions themselves.

Similarly, the way in which X modulates variability (as based on x) is also decomposed into subprocesses affecting different parts of the agent differentially. If x is low because a specific trait is malfunctioning, variability need not (and will not in general) be redirected to that specific trait. How variability is redirected and distributed in specific organisms is likely to be quite complex, depending on the particulars of the organism and its habitat. However, the way in which X distributes variability is readily evolvable through standard evolutionary mechanisms, because it affects f. It is therefore likely to be adequate, on average. As an example of how variability may be redirected, we can consider the function of hemoglobin in vertebrates. It has the function of enhancing oxygen transport, according to existing theories of biological function. The new theory ascribes this function to hemoglobin as well, as follows. If hemoglobin starts to work less effectively, such as in the presence of interfering chemicals, then this is detected by control circuits regulating the oxygen levels in an organism. Compensatory changes (e.g., to respiration) are then made through standard feedback control, primarily in a deterministic way. The new theory conjectures that a deficient oxygen level produces, in addition, effects through X. This is done in a stochastic way and is based on approximating (estimating) the organism’s overall fitness. The oxygen level is one of the factors likely to be used for producing such an overall fitness estimate, because this level is highly significant for the actual fitness. Therefore, X has likely evolved to include it, because that improves the adequacy of x as an estimator of fitness. Therefore, a poor performance of hemoglobin reduces x, and thus, indirectly, drives more variability anywhere in the organism. For example, it may result in behavioral variations that eventually result in the organism finding a less energetic lifestyle. Such a lifestyle can enable it to survive, despite suboptimal oxygen levels. The new lifestyle can become fixed (through a reduction of behavioral variability), because X subsequently indicates that the expected (i.e., approximated) fitness has become fairly high again.

In conclusion, biological functions can acquire ontic-causal status as follows. If a trait, process, or behavior is of evolutionary significance to an agent, for example the pumping of blood by the heart, then it is likely to be represented in X. This is likely, because X would need to monitor the blood circulation in order to produce an x that is a reasonable approximation of f. A poorly working blood circulation should be reflected in a decreased x. A reasonable approximation of f by x is required for obtaining high fitness (through the mechanism of the G loop). It is therefore under positive selection pressure. We have seen above that subprocesses of X have autonomous causal efficacy, that is, they are ontic-causal. Therefore, the function as such is also ontic-causal. It has a non-material causal aspect (through X) that occurs in addition to the material realization of the function itself (such as is realized by the heart and its muscles).

In order to decide whether a trait or process is functional in the ontic-causal sense, one needs to determine whether it is represented in X, that is, whether it is monitored by X (and thus used for producing x and for modulating organismal variability). Whether a trait or process is monitored by X is ultimately an empirical question. X is just a physiological or neural process that can be identified and modeled, including if and how it tracks the performance of specific traits or processes. If X exists (as conjectured here), it must be included in any adequate model of the organism. When a good model of X is established, then this also establishes what is represented in X and what not.

Until such empirical and modeling studies are available, common sense arguments may be used to evaluate the proposal made in this article (see, e.g., the section “Intuitions About Functions”). The key notion here is that X itself has evolved and is subject to continuing evolutionary pressure. If x approximates f well, it gives the organism an evolutionary advantage. But like any biological process, X is costly (e.g., in terms of energy and material use), thus it will typically acquire parts that are useful and, eventually, lose parts that have become useless. Moreover, useless parts may even reduce how well x approximates f. Such a reduction would decrease the organism’s evolutionary advantage, because it would decrease how well the G loop works. Useless parts in X would, then, be specifically selected against. Thus, one can use the usual evolutionary reasoning to make plausible arguments as to what is included in X and what not.

A provisional definition that may be useful for such commonsense arguments is that “the working of a biological trait or process has an ontic-causal function if and only if its performance is monitored by X—where how X implements the sign of the trait’s contribution to x determines how one should formulate the function.” It is important to note that monitoring as such is neutral with respect to the question whether the effects of a trait or process in specific cases contribute positively or negatively to x. The mere fact of being included in X is already sufficient for having an ontic-causal function. Therefore, a malfunctioning heart still has the function of pumping blood, because its performance continues to be monitored by the X process. Nevertheless, the implemented sign of the contribution to x is important for how one should, linguistically, formulate the function. Saying that the function of the heart is “to pump blood” is correct, because “pumping blood” is implemented in X in such a way that it contributes positively to x (and thus is an implicit goal). One might perhaps interpret “monitoring pumping blood” alternatively as “monitoring not pumping blood.” But saying that the function of the heart is “not to pump blood” is incorrect, because “not pumping blood” contributes negatively to x (and thus is not a goal, but something to be avoided). The definition explicitly includes “ontic-causal,” because one is free, of course, to define biological functions more broadly, i.e., in an epistemic-real sense. A broadly defined concept of function may be convenient when used metaphorically in certain scientific explanations, even if it assigns functions to processes that have no autonomous ontic-causal status.

Ideally, functional goals represented in X would always serve f, because x is under selection pressure to approximate f as well as possible. However, this is not guaranteed, and agents may therefore have goals that are not in their best interest. Such goals can only be transient, because they are selected against or found to be disadvantageous through learning, eventually. Therefore, x tends to be well aligned with f.

## Other Theories of Functions

Broadly speaking, there are two main traditions for explaining biological functions. The causal role (CR) school (Cummins 1975, 2002) characterizes functions by their current causal role in accomplishing assumed capacities of a containing system. In biological organisms, such capacities may take the form of specific goals, e.g., survival and reproduction (Boorse 1976). In contrast, the selected effects (SE) school (Millikan 1984, 1989; Neander 1991) looks at the historical, evolutionary causes of biological functions. Although some approaches incorporate elements of both schools (e.g., Walsh and Ariew 1996; Buller 1998) and there are alternative approaches, I use a clean dichotomy here for explanatory purposes. This clearly exposes the problems that arise if one seeks to assign ontic-causal status to biological functions.

### Selected Effects Functions

**Fig. 2:**
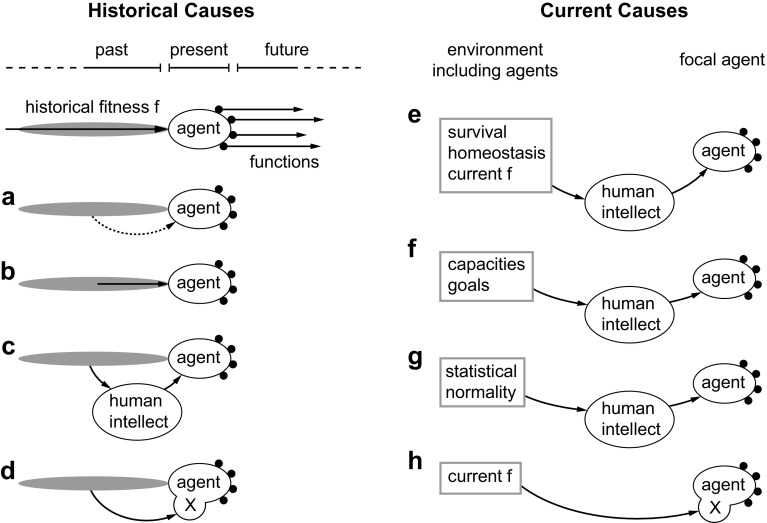
Theories of biological function are typically based on historical, evolutionary causes (*left*) or current causes (*right*)

The upper diagram in the left part of Fig. 2 illustrates the basic idea of the SE explanation. This explanation is also known as etiological, that is, with the explanation provided by a chain of historical causes. A particular agent has functions that are active, or at least potentially active, in the present or future (black dots and arrows). The SE approach assumes that these functions can be explained by their origin, through natural selection, in the evolutionary past of an agent (Millikan 1984, 1989; Neander 1991). Alternatively, such selection can be formulated in terms of fitness (Griffiths 1993; Buller 1998), by requiring that functions have contributed positively to the fitness of the agent’s ancestors. In the figure, this is symbolized by the historical fitness f. Either way, natural selection and the effects of fitness occurred in a distributed way over time, which is symbolized by the gray area.

As stated above, we seek to assign ontic-causal status to functions. Functions exist in the present. If they are ontic-causal partly because of a historical process (historical fitness), then the question arises how this historical process is connected to the current entity. Some causal connection must be present if functions are to be ontic-causal. Without such a connection, the functions could only be epistemic-real. The main possibilities I can think of that might produce such a causal connection are depicted schematically in Fig. 2a–d.

The first possibility (Fig. 2a) assumes that there is a causal connection through immaterial (e.g., Platonic) means (dotted arrow). For example, one may assume that a historical process consists of objective facts, that it exists in its own right, and that it extends its existence across time (similar to a Platonic circle, which could be seen as timeless). It can then connect to the present function. However, such an immaterial explanation has no clear naturalistic interpretation. It seems too implausible to be considered further here.

The second possibility of a causal connection (Fig. 2b) is the standard way by which causal influences are thought to be connected to one another in physicochemical processes. Such processes are fully defined by an instantaneous state, at each moment in time, that proceeds to the next state, at the next moment in time. Importantly, such processes do not contain explicit information about earlier states. This lack of historical information implies that the mechanism of Fig. 2b cannot directly connect the relevant parts of the fitness history to the present. At most, it only transfers information about the state immediately preceding the present one. Everything before is “forgotten” and irrelevant from a physicochemical point of view, because physicochemical states unfold locally in time. There is no way to tell, purely from the state, how the system got to that state. Its history can only be reconstructed by using specific background information. But that would be epistemic inference. Relying on it would only produce functions with epistemic-real status.

A variant of the causal connection of Fig. 2b was proposed by Millikan (1984). Lines of descending organisms are connected by an uninterrupted chain of reproduction. Reproduction thus transfers the effects of natural selection (or of fitness) across time. However, reproduction has no special status from a naturalistic point of view. It is just a physicochemical process that is completely defined by processes unfolding locally in time. In other words, nothing is transferred beyond the immediate physicochemical state.

One might think that developmental processes in an organism can solve the problem of causally connecting the present function to the evolutionary past, because they construct a trait as homologous to ancestral traits. However, such an explanation depends on epistemic interpretation. It requires human perception to note the structural correlation that is associated with “homologous.” Such a correlation has no autonomous causal efficacy. It can be a factor in scientific explanations, but it does not belong to the fundamental causal inventory of the world. Thus, functions explained in this way are only epistemic-real.

Similarly, nothing is solved if one would invoke DNA as a carrier of historical information. Biological functionality is used already when one interprets DNA as a form of memory. Memory presupposes biological functionality, because it assumes that it is possible to refer across time. Conventional physicochemical processes cannot refer across time or space, because all interactions are strictly local in time and space. In contrast, the theory explained above can produce nonlocal causation because of the nonlocal reference that x makes to f. However, this already requires agents that are subject to evolutionary pressure and that possess an X system (this is formalized mathematically in van Hateren 2015c). Fundamentally, nonliving physicochemical processes lack memory (the memory in machines is a macroscopic phenomenon that presupposes human interpretation; at the microscopic level, machines do not utilize memory). Using memory for explaining the ontic-causal status of biological functions would be circular, unless one first explains nonlocal reference across time (by introducing X).

The scope of memory is evolutionary in the case of DNA, but the problem remains for faster forms of memory. For example, Garson (2012) proposes a generalized selected effects theory for functions in neural systems, utilizing selective (but non-evolutionary) processes acting on synapses, neurons, or neural groups (e.g., through development and learning). However, selected neural functions are formed at an earlier moment than when they are typically used. In other words, the causation would depend on memory, and would be epistemic-real again. Therefore, it would fail to give ontic-causal status to functions.

The current argument is similar to the intuition inherent in well-known counterexamples against SE theory. Such counterexamples involve organisms that are identical to actual ones but with a completely different history, such as hypothetical instant organisms (e.g., Swampman) that are produced spontaneously (Boorse 1976, p. 74; Neander 1996; McLaughlin 2001, pp. 108–113). If functions have an ontic-causal status and naturalism is true, identical organisms must have identical functions. But according to basic SE theory, different histories would imply different functions. Therefore, basic SE theory must be amended if one seeks to assign ontic-causal status to functions (see below).

The third possibility of causally connecting history with present functions is sketched in Fig. 2c. It involves human intellect interpreting the fitness history of a specific agent and assigning functions to the appropriate processes. The historical information that was lacking in Fig. 2b is now implicitly present in human intellect and memory. Human intellect thus connects historical fitness to the present agent. However, intellect already presupposes biological functionality, because it depends on memory, agency, goal-directedness, and nonlocal reference in general. The possibility of Fig. 2c is perfectly legitimate and is standardly used for scientific inference. But it only produces functions that are epistemic-real. The function ascription is objective and real for the human (in the sense of the real patterns of Dennett 1991), but it does not produce an ontic-causal function in the agent.

The final possibility of producing a causal connection between fitness history and functions (Fig. 2d) assumes a special process in the agent, X. As explained above, this can indeed produce ontic-causal functions. Information on the fitness history is implicitly stored in the structure of X. Part of the theory can be seen as an amended version of SE theory, where x, rather than f, is utilized (see below).

### Causal Role Functions

The right half of Fig. 2 illustrates several variants of the CR explanation. This theory focuses on the present and investigates the causal role that functions have for the current capacities of an agent. Such capacities are typically relative to the agent’s internal state and to its environment, including other agents. Clearly important to an agent are the capacities to survive, to maintain homeostasis, and to obtain a high fitness f. However, these are compound factors, which do not have causal efficacy beyond that of their constituent factors. For example, the fitness of a bacterium is produced by a multitude of physical factors (temperature, presence of nutrients, absence of antibiotics, and so on). Only these factors directly influence the bacterium and its chances of survival and reproduction. In contrast, fitness itself is an epistemic-real factor. Fitness is objective and real, and plays an important role in human scientific theories. But it has no autonomous causal efficacy beyond that of its constituents and their interactions, and it cannot make functions ontic-causal. Therefore, functions acquire mere epistemic-real status if they are explained by their role for survival, fitness, and homeostasis. Human intellect is then required (Fig. 2e).

Capacities (Cummins 1975) or goals (Boorse 1976) are explicitly assigned during human analysis of a system (Fig. 2f). They depend on the causal organization of the system. However, “organization” is an epistemic-real phenomenon, not an ontic-causal one. Inferring organization is part of human functionality. Organization in abiotic systems never has causal efficacy of its own, even if such systems are complex. For example, there appears to be structure and organization, in the form of nonlocal correlations, in the atmospheric system that produces weather and climate. Scientific theories about the atmosphere depend on specifying this structure. They may use complex explanatory factors in the form of correlated aggregates, such as clouds, tornadoes, seasons, and ice ages. But such structure has arisen gradually and naturally from the history of system states, without structure itself participating in the causal dynamics. The actual causation is purely local, through local pressure, local radiation, local mass transport, and so on. Only those local factors are needed in the fundamental causal inventory of the world.

According to the standard naturalistic view of living organisms (i.e., without conjecturing an X process), they are also just physicochemical systems, albeit highly complex ones. They may be more complex than most abiotic systems, but they are still fully driven by the standard local causation of any physical and chemical process. Nevertheless, living organisms appear special, because they have a cyclically closed organization. This forms the basis of organizational accounts of function (Mossio et al. 2009; Moreno and Mossio 2015). In a closed organization, the system specifically produces products and conditions that are required for sustaining the working of the system itself. This also happens in some simple abiotic systems, such as a candle flame (which sustains itself by drawing in its own fuel and oxygen). But living organisms do this in ways that are far more differentiated and complex. However, one can still completely define the dynamics of a complex cyclical system in terms of the local processes and local interactions of which the system is composed. Its complexity does not make it fundamentally different from the atmospheric system. One could specify all molecular components and interactions of a metabolic system in a similar way as those of the atmosphere, and readily simulate either system. In other words, “organization” need not be included in a fundamental causal inventory of the world. It has no autonomous causal efficacy, neither in a candle flame, nor in a standard (X-lacking) living organism. It cannot give ontic-causal status to biological functions.

In contrast, living organisms that contain an X process do have an additional causal factor that goes beyond the standard causation of abiotic systems. The presence of X in a G loop introduces a relation as a causal factor, namely the approximating relationship between x and f. This relation cannot be reduced to local processes and local interactions. Moreover, X and x integrate processes across the organism, both by affecting and by being affected. This provides the organism with a form of unity that is lacking in abiotic processes. In abiotic processes, one can always eliminate structure as a causal factor, as in the weather and climate example given above. But this eliminative strategy does not work in the case of living organisms that contain an X process. Elimination would leave no room for the relation between x and f. It would thereby neglect an essential, ontic-causal part of how living organisms work. Living organisms are, therefore, intrinsically distinct, non-epiphenomenal entities, in contrast to, e.g., a tornado. X is evolvable, and could gradually emerge from systems lacking X. Therefore, the theory does not assume a property (distinctness) in order to explain that property. The explanation involves gradual change through time, which makes the explanation cyclical rather than circular. It is therefore perfectly legitimate. Finally, it should be recognized that both organismal unity and causally efficacious relations are key notions of the organizational theory of functions (see, e.g., Moreno and Mossio 2015, Chap. 2). The current theory may be viewed as providing a naturalistic grounding of such notions.

One way to detect what functions are typically doing is to observe the distribution of their properties in a population (Fig. 2g). This yields an estimate of statistical normality (Boorse 1977). The distribution of properties in a population approximately reflects the evolutionary history of the function, in that it is likely to be concentrated at properties that contribute positively to fitness. Therefore, current statistical normality can be regarded as the population version of the historical SE approach. However, distributions of properties have no autonomous causal efficacy and cannot directly influence organisms. Such distributions are epistemic-real entities, not ontic-causal ones. This approach, therefore, produces epistemic-real functions, depending on human intellect (Fig. 2g). The recent modal theory of Nanay (2010) also requires human intellect, because it depends on inferring the effect of functions in “relatively close” possible worlds. Possible worlds are entities that cannot exert direct causal influence, and thus can only be used for explaining functions as epistemic-real.

As before, the only way to avoid human intellect is through an internal process X within the agent (Fig. 2h). X refers, implicitly, to the relevant factors in environment and agent. Functions become ontic-causal because of the causal efficacy of the relation between x and f.

## Unification of Theories of Biological Function

As argued above, the ontic-causal efficacy of functions derives from the fact that x approximates f. X is itself an evolved physiological or neural process. Therefore, the history of f has shaped the way in which X lets x approximate f. Thus, the structure of X depends on that history. It is, therefore, closely associated with the selected effects theory of functions. When X is used for explaining functions, the history of f is used as well, albeit only implicitly and indirectly (Fig. 2d). The implicit memory of X that appears to be present here does not presuppose biological functionality (in contrast to when one would directly invoke developmental or genetic memory). It has emerged naturally from the evolved property of X that x approximates f (and that parts of X approximate corresponding parts of F).

**Fig. 3:**
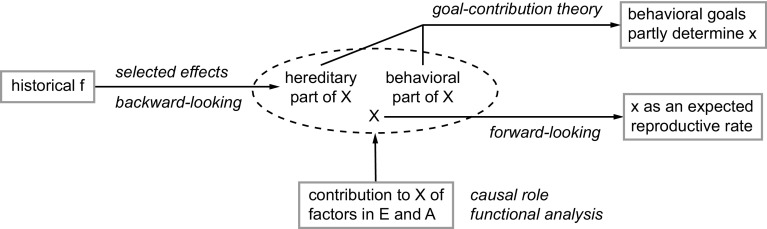
The internalized process approximating fitness, X, can serve as an anchor point for amended versions of most previous theories of biological function

In addition to the part of X that focuses on heredity and fitness history, there is also a behavioral component in X (Fig. 3). This component modifies the organism during its lifetime, through phenotypic plasticity and similar processes. Again, these modifications depend on the requirement that x approximate f. There is no certain way for the organism to verify, on the spot, the correctness of this approximation. But effective mechanisms to that end must have evolved over evolutionary time. For example, learning strategies must have evolved that are likely to produce adequate approximations, on average. The behavioral part of X has no direct selected effects explanation (unless the concept of selection is stretched, as in Garson 2012). It is particularly associated with the causal role theories of function (Fig. 2h), because it specifically attempts to track real-time changes in F and f. The behavioral part of X is continually adjusted during the lifetime of an organism. Capacities and goals can thus become part of X.

X is causally effective because of two different causal aspects that are both necessary, as was explained above: first, a nonconventional causal aspect in the form of an approximating relationship between x and f; second, a conventional material causal aspect in the form of the physicochemical realization of X. The latter is a conventional process that monitors the condition of the organism and affects its variability. Functions are fully defined by how X monitors. In other words, functions do not depend on the etiology of X, but only on the current structure of X. Organisms that arise spontaneously (e.g., Swampman) have exactly the same X as identical evolved organisms, and they have therefore exactly the same functions. Neither does the causal efficacy of X depend on its etiology. Given identical organisms in identical circumstances (now and in the future), F and f will be identical, as well as the relation between x and f. X will then have the same effects on Swampman as on its natural counterpart. Nevertheless, etiology is still needed for understanding how X and its structure could arise.

The above considerations suggest that replacing f by x (and F by X) in existing theories of function has two major consequences. First, it aligns these theories with specific aspects of the new theory. Second, the existing theories will then actually produce ontic-causal rather than epistemic-real functions (Fig. 2d, h). This follows from the fact that X has autonomous causal efficacy, whereas f does not. One way to state the novelty of the present proposal is by noting that earlier accounts only consider the direct material realizations of functions (e.g., how they work or how they have been formed by natural selection). In the new account, natural selection works, in addition, on the X process. The X process monitors, but it does not directly (i.e., immediately and proximately) participate in the working of functions. X only indirectly affects functions, by modulating how much they can vary (and thus how fast they can change, potentially). The material realizations of functions do not require relations as causal factors (similarly to the fact that the weather does not require relations). In contrast, the ultimate causal efficacy of X does require relations (similarly to a weather simulation, which depends on relations with the actual weather if it is to be accurate and useful).

A taxonomy of existing theories of biological function is provided by Perlman (2004, 2009). The three main branches of that taxonomy are nonnaturalistic theories (Platonic and religious), quasi-naturalistic theories that depend on the notion of emergence, and naturalistic theories. The latter theories are subdivided into conventionalism and theories that are primarily backward-looking, present-looking, or forward-looking. I will focus here on the latter three. Figure 3 illustrates that they can be viewed as representing different aspects of the new theory, by reformulating them within the new framework (by using X and x, rather than F and f).

The reformulation of the selected effects theory focuses on functions with goals related to the hereditary part of X. This part is formed by the evolutionary history of f in an agent’s lineage (leftmost arrow). That part of X can be regarded as backward-looking (in accordance with Perlman’s classification), because the structure of X implicitly refers to the evolutionary history. The reformulations of causal role theories (present-looking in Perlman’s classification) specify how the factors of environment E and agent A contribute to X and its subgoals (Fig. 3, upward-pointing arrow). Formally, biological functions can then be regarded as capacities that are expected to realize the present subgoals of X. This realization involves mechanisms using factors in E and A, as sensed by the organism in the present (Fig. 1, right part).

Goal-contribution theories (e.g., Boorse 1976) depend on current goals of an agent. Perlman classifies them as backward-looking to the recent past. Such theories can also be reformulated within the new framework. The current goals may then have been established recently in the hereditary part of X. Alternatively, they can belong to the behavioral part of X when they are acquired during the lifetime of an agent, such as through learning. The upper rightmost arrow, originating from both parts of X, symbolizes the rationale of these theories. Finally, forward-looking approaches (e.g., Bigelow and Pargetter 1987) focus on the overall goal of obtaining high f. When reformulated within the present framework, they focus instead on the overall goal of obtaining high x (which is in fact a true goal, in contrast to obtaining high f, which is only an “as if” goal).

Figure 3 shows that these previous theories can be positioned in the new theory, although always with an essential and obligatory switch from f to x. The new theory unifies the earlier ones, and adds their explanatory power (see the next section). All causation in Figs. 1 and 3 involves well-understood forms of causation, either primarily deterministic, primarily random, or combinations. The theory is therefore fully naturalistic. The required mechanisms are evolvable through standard natural selection (van Hateren 2015a). Nevertheless, the special, nondeterministic G loop, as depicted in Fig. 1, produces a unique, emergent goal-directedness. This arises from the unusual fact that a relation, namely the one between x and f, has acquired autonomous causal efficacy.

## Intuitions About Functions

Based on an extensive literature review, Wouters (2005) compiled a list of 15 intuitions about functions with which a theory of functions should ideally comply. He concluded that no existing theory could handle them all. Below they are discussed from the perspective of the new theory (all quotations are from Wouters 2005, pp. 133–134). The arguments rely on the fact that X itself has evolved, and that it continues to change on evolutionary and behavioral timescales. It gives the organism an evolutionary advantage only if x is a reasonable approximation of f. Therefore, X will typically contain and acquire components that are useful for such an approximation, and lose those that have become useless or detrimental.

1. “A theory of function should distinguish between activities that are functions (such as the beating of the heart) and activities that are side-effects of functional organs (such as heart sounds and pulses).” Side-effects are not included in the hereditary part of X (as they played no role in evolving X) and are therefore not automatically functional. However, when a side-effect is incorporated into the behavioral part of X, through learning, it may become functional.
2. “A theory of function should not allow one to ascribe functions to parts of systems that are not believed to have parts with functions (such as our solar system).” The solar system is not a living organism. It has neither f nor X, and therefore no parts with functions.
3. “A theory of function should allow for maladapted functions.” The fur of a polar bear has as its primary function the reduction of heat loss. This function is determined by the hereditary part of the bear’s X (as heat loss is of such importance for fitness that X must have evolved to utilize it for making x an adequate estimate of f). However, when the bear lives in a zoo in the tropics, f deviates from x (and the corresponding parts of F deviate from the corresponding parts of X). The fur is then maladaptive because it lowers f, but it is still a function for the bear because it remains incorporated in the bear’s X.
4. “A theory of function should not depict the use other organisms make of the items of a certain organism as functions of those items. It is, for example, not a function of a dog’s long hair to harbor fleas.” For the dog, using its long hair for harboring fleas is not a function, because it is not incorporated in the dog’s X as a goal, i.e., as a factor that increases x. For the flea, living in the long hair of a dog is likely to be incorporated in the flea’s X as a goal.
5. “A theory of function should distinguish between effects that are functions and effects that are accidentally useful. Although belt buckles occasionally save their wearers’ life by deflecting bullets, it is not a function of belt buckles to deflect bullets.” Accidentally useful effects just happen to contribute to f. But they are not incorporated in X (as they played no role in evolving X), and they are therefore not functions.
6. “A theory of function should not depict the systematic use humans make of existing items for new purposes as functions of those items. It is, for example, not the function of the human nose to support eyeglasses.” It is not the default biological function of the nose, because it is not included in the hereditary part of X (as eyeglasses played no role in the evolution of X). Only when X is adjusted through learning, the nose may acquire an additional (though learned rather than biological) function for an agent.
7. “A theory of function should allow one to attribute functions to traits that currently do not vary in the population.” The theory only requires that traits are expected to contribute positively to x and thereby probably to f. A positive contribution to fitness may not be observable in population variability. For example, some functions may play such a fundamental role for cellular functioning that any genetic variation in them would be lethal. Such variations are nevertheless bound to happen (for molecular reasons), but would not produce viable cells. They would therefore not be observable as phenotypic variation in a population.
8. “A theory of function should distinguish currently functional items from vestiges (like vestigial eyes in cave dwellers).” Vestigial eyes in cave dwellers are likely to have lost their representation in X, because if they were still included then that would lower the accuracy by which x approximates f. Thus, it would have been selected against in previous evolution. Without representation in X, such eyes have no function for cave dwellers.
9. “A theory of function should allow one to attribute functions to the parts and behaviors of so-called ‘instant organisms’, hypothetical organisms that have no evolutionary history.” Instant organisms are created including their X. X is just a concurrent physiological process. Those parts and behaviors that it monitors are functional. This is the same in an instant organism as in an identical organism with another history. Thus, the former has the same functions as the latter.
10. “A theory of function should enable us to attribute functions to items that do not actually perform it (most sperm cells will never fertilize an egg cell and mating displays quite often do not have the intended effect).” Functions correspond to subgoals of X, which are, like X itself, to be understood in a probabilistic sense. They are expected to contribute, on average, to x and therefore, probably, to f. Sperm cells are indeed likely to contribute to f, statistically. Most do not, but the few ones that do are highly significant for fitness.
11. “A theory of function should enable us to attribute functions to items such as malformed hearts that are incapable of performing their function.” A malformed heart influences only f, not the inclusion of its functional goal in X (which was established when X evolved). Therefore, it retains its function, even when X and x indicate it is malfunctioning. The same applies to the case when epidemics and major disasters reduce f in an entire population. Functions only depend on the form of X, and they are therefore not changed by epidemics.
12. “A theory of function should allow one to attribute functions to the parts and behaviors of sterile organisms such as mules.” Mules have a normal X and thus have the usual functions.
13. “A theory of function should not allow one to attribute functions to organisms as a whole.” Organisms as a whole could only have a function if they are part of a larger system that has f and X. In that case, they would have a function for that larger system, not for themselves. One possible candidate for such a larger system is an ecological system. But such a system does not have a clear reproductive rate (required for f), and there are no indications that anything resembling X and a G loop could be present in an ecological system. A larger system that perhaps might have f and X is a colony of social insects (briefly discussed in van Hateren 2013). Animal husbandry is a clear case where organisms as a whole can indeed have a function, e.g., when keeping sheep for their wool is incorporated into the behavioral part of human X. But sheep are then merely functional for humans, not for themselves.
14. “A theory of function should not allow one to attribute functions to such things as junk DNA, selfish DNA, and segregation distorter genes.” Junk DNA and other forms of DNA that do not contribute to f are unlikely to have their working monitored by subprocesses of X. If X would implicitly attribute x-enhancing effects to such forms of DNA, the approximation of f by x would be less accurate. Therefore, it would be selected against.
15. “A theory of function should allow one to attribute functions to traits that are selected against.” Circumstances may have changed such that not having a specific evolved trait, or having another trait, produces higher f. The trait is then selected against. But it may still be relevant for producing an x that approximates f, and therefore still be monitored by X (and thus be functional). There will be growing selection pressure on X to stop monitoring a trait if the trait gradually disappears or becomes irrelevant for f.

It is clear that the new theory performs very well. All intuitions are aligned with the explanations of the theory. Yet, the original theory (van Hateren 2015a) was not explicitly intended for explaining intuitions about biological functions. In that sense, the correspondence shown above is a successful prediction of the theory.

## Discussion and Conclusion

The analysis in this article makes it plausible that biological functions can indeed have an ontic-causal status. This requires a physiological process X within an agent that produces an approximation of the agent’s actual fitness, f. The intrinsic X participates in a causal loop that is evolvable and sustainable by conventional evolutionary mechanisms. The loop produces genuine agency and goal-directedness in living organisms, and makes the goal-directedness and normativity of functions ontic-causal as well. This ontic-causal status requires that functions in an agent be represented in X. Processes contributing to f without being monitored by X might be perceived by an observer as adaptations. They could be perceived as functional in the sense of objectively contributing to the agent’s fitness f. However, such functionality would only be epistemic-real. It would only play a role for human scientific understanding. The agent itself is only directly connected to X, not to f and its history. Therefore, only functions that are included in X are ontic-causal. Only those functions strictly exist as autonomous, goal-directed parts of the causal dynamics of the agent.

Functions based on X combine the historical view of selected effects theories with the ahistorical view of causal role theories. The reason is that X forms, in effect, an implicit memory of previous evolutionary outcomes. In addition, it is adjustable in the present through learning and phenotypic plasticity. On the one hand, it is backward-looking to the distant and recent past. On the other hand, it is present- and forward-looking, because fitness is associated with the current likelihood of surviving and reproducing.

The theory presented here is new and largely conjectural. Nevertheless, there are strong reasons to think it is a plausible one. First, there are computational reasons; second, theoretical reasons; third, it can explain and unify a wide range of phenomena; and fourth, it is consistent with mounting evidence for the role of randomness in living organisms. Computationally, simple models show that the mechanism presented in Fig. 1 not only works, but also is evolvable for a range of conditions and models (van Hateren 2015a). The mechanism is advantageous, is quite simple in simple organisms, and requires only a slight variation on existing mechanisms. It is therefore plausible that evolution has produced it, at or close to the origin of life (van Hateren 2013). The mechanism uses modulated randomness as an essential causal factor. The proposed system critically depends on and is evolvable through evolution by natural selection. This makes it understandable why such properties can only be observed in living systems.

As shown in the previous section, the theory is quite successful in explaining intuitions about biological functions. Moreover, it largely matches with the concept of meaning in biological systems that has been developed in the field of biosemiotics (van Hateren 2015d). It explains why life seems to be characterized by having agency (van Hateren 2013, 2015a). Other examples could be added. Many of these applications of the theory concern topics where alternative theories are absent, problematic, or only partially successful. A theory that can integrate wide, seemingly disconnected parts of reality in a well-defined way has intrinsic plausibility. Even if its components have not yet been shown explicitly, the fact that the theory has considerable explanatory power adds to the likelihood that such components actually exist.

Finally, there is mounting empirical evidence for the importance of functional randomness in living systems (Faisal et al. 2008; Brembs 2011; Kiviet et al. 2014). Several studies provide circumstantial evidence for the specific mechanism of Fig. 1. At the subcellular level, mutation rates are known to be modulated in proportion to cellular stress (Galhardo et al. 2007), with stress presumably inversely related to cellular x. At the cellular level, the run-and-tumble behavior of the bacterium *E. coli* (Macnab and Koshland 1972) provides an example of randomness modulated by the availability of nutrients, also associated with fitness. At the neural level, a similar modulation of turning rates and randomness has been shown in the nematode worm *C. elegans* (Gray et al. 2005; Gordus et al. 2015). In the context of foraging behavior, switching from local search to a wider search area when the yield of food patches becomes low appears to follow a similar pattern in many species (Hills 2006). Neural plasticity as controlled by how dopamine depends on reward prediction errors (Glimcher 2011) seems to conform as well. The dopaminergic system may thus contribute to X, at least partly.

However, all such examples may have alternative explanations, and their precise role for fitness is not clear. Ultimately, only targeted experiments with associated theoretical modeling can provide conclusive evidence for the theory. X is conjectured to integrate information about much of what is going on in an organism, and to produce effects throughout the organism. Therefore, a comprehensive system-theoretic understanding of the entire organism is required. Quantitative evaluation is probably only practicable, then, in very simple organisms. Nevertheless, there is no reason why empirical testing could not be performed, even if it would require considerable effort.
